# Effect of COVID-19 Pandemic on Acute Coronary Syndrome Clinical Practice Patterns: Findings from a Multicenter Clinician Survey in China

**DOI:** 10.31083/j.rcm2311362

**Published:** 2022-10-25

**Authors:** Feng Hu, Minhua Zang, Lihui Zheng, Wensheng Chen, Jinrui Guo, Zhongpeng Du, Erpeng Liang, Lishui Shen, Xiaofeng Hu, Dezhong Zheng, Xuelian Xu, Gaifeng Hu, Aihua Li, Jianfeng Huang, Yan Yao, Jun Pu

**Affiliations:** ^1^Department of Cardiology, Renji Hospital, School of Medicine, Shanghai Jiaotong University, 200030 Shanghai, China; ^2^Department of Cardiology, Fuwai Hospital, National Center for Cardiovascular Diseases, Chinese Academy of Medical Sciences and Peking Union Medical College, 100730 Beijing, China; ^3^Department of Cardiology, Guangdong Provincial Hospital of Chinese Medicine, 510120 Guangzhou, Guangdong, China; ^4^Department of Cardiology, Fuwai Yunnan Cardiovascular Hospital, 650102 Kunming, Yunnan, China; ^5^Department of Cardiology, Zhu Jiang Hospital of Southern Medical University, 510260 Guangzhou, Guangdong, China; ^6^Heart Center of Henan Provincial People's Hospital, Central China Fuwai Hospital, Central China Fuwai Hospital of Zhengzhou University, 451460 Zhengzhou, Henan, China; ^7^Department of Cardiology, Affiliated Hangzhou First People's Hospital, Zhejiang University School of Medicine, 310030 Hangzhou, Zhejiang, China; ^8^Department of Cardiology, Shanghai Chest Hospital, Shanghai Jiao Tong University, 200030 Shanghai, China; ^9^Department of Cardiology, The Third Affiliated Hospital of Southern Medical University, 510630 Guangzhou, Guangdong, China; ^10^Department of Cardiology, University-Town Hospital of Chongqing Medical University, 401331 Chongqing, China; ^11^Department of Cardiology, The First Affiliated Hospital of Wenzhou Medical University, 325035 Wenzhou, Zhejiang, China; ^12^Department of Cardiology, The Affiliated Hospital of Yangzhou University, 225003 Yangzhou, Jiangsu, China

**Keywords:** COVID-19 pandemic, acute coronary syndrome, ST-segment elevation myocardial infarction, non-ST-segment elevation myocardial infarction, unstable angina

## Abstract

**Background:**

The coronavirus disease 2019 (COVID-19) pandemic has severely affected healthcare systems around the world. This study aimed to investigate the perceptions of cardiologists regarding how the COVID-19 pandemic has affected the clinical practice patterns for acute coronary syndrome (ACS).

**Methods:**

A multicenter clinician survey was sent to 300 cardiologists working in 22 provinces in China. The survey collected demographic information and inquired about their perceptions of how the COVID-19 pandemic has affected ACS clinical practice patterns.

**Results:**

The survey was completed by 211 (70.3%) cardiologists, 82.5% of whom were employed in tertiary hospitals, and 52.1% reported more than 10 years of clinical cardiology practice. Most respondents observed a reduction in ACS inpatients and outpatients in their hospitals during the pandemic. Only 29.9% of the respondents had access to a dedicated catheter room for the treatment of COVID-19-positive ACS patients. Most respondents stated that the COVID-19 pandemic had varying degrees of effect on the treatment of acute ST-segment elevation myocardial infarction (STEMI), acute non-ST-segment elevation myocardial infarction (NSTEMI), and unstable angina. Compared with the assumed non-pandemic period, in the designed clinical questions, the selection of coronary interventional therapy for STEMI, NSTEMI, and unstable angina during the COVID-19 pandemic was significantly decreased (all *p *< 0.05), and the selection of pharmacotherapy was increased (all *p *< 0.05). The selection of fibrinolytic therapy for STEMI during the pandemic was higher than in the assumed non-pandemic period (*p <* 0.05).

**Conclusions:**

The COVID-19 pandemic has profoundly affected ACS clinical practice patterns. The use of invasive therapies significantly decreased during the pandemic period, whereas pharmacotherapy was more often prescribed by the cardiologists.

## 1. Introduction

The coronavirus disease 2019 (COVID-19) pandemic, caused by severe acute respiratory syndrome coronavirus 2, has rapidly spread and has resulted in considerable morbidity and mortality [1, 2, 3, 4]. The pandemic has severely affected healthcare systems around the world, and these systems have struggled to effectively prevent and treat COVID-19. Furthermore, there has been additional pressure because the pandemic has profoundly disrupted the clinical practice patterns of other common diseases.

The management of acute coronary syndrome (ACS) during the COVID-19 pandemic should be investigated to better understand the balance between the clinical benefits of treatment and the risk of viral transmission. Previous studies have found a significant increase in the morbidity and mortality of ACS patients owing to direct and indirect effects of the COVID-19 pandemic [5, 6, 7, 8, 9, 10, 11]. Although there has been an expert consensus on the interventional treatment of acute myocardial infarction during the COVID-19 pandemic [12], decision-making for ACS treatment during the pandemic has been problematic owing to different and unequal medical resources around the world. In particular, many hospitals do not have dedicated cardiac catheterization rooms for COVID-19 patients. At present, there is a lack of real-world data from large-sample randomized controlled trials to understand how COVID-19 has affected the clinical practice patterns of ACS. In this study, we conducted a questionnaire survey of cardiologists from 22 provinces in China to investigate their views on how the COVID-19 pandemic has affected their ACS treatment strategies.

## 2. Methods

### 2.1 Study Design and Participants

The intended target population of the survey was cardiovascular department physicians who treat patients with ACS. An online survey was distributed via WeChat (Version 8.0.21, Tencent, Shenzhen, China) software to 300 cardiologists in 22 provinces in China (**Supplementary Fig. 1**) between April 1, 2022 and April 30, 2022. The clinician survey was completed anonymously, and all responses were submitted by April 30, 2022. The study was approved by the Ethics Committee of Renji Hospital affiliated with the School of Medicine, Shanghai Jiao Tong University (KY2022-096-A).

### 2.2 Survey Design

The survey was administered in Chinese and consisted of three parts (translated into English and available in **Supplementary Material**). In part 1, we collected the participants’ demographic information, including years of practice, subspecialty, hospital level, and province. Part 2 included questions regarding the severity of the COVID-19 pandemic in their province. Part 3 consisted of structured questions to gain insight into how the COVID-19 pandemic affected their ACS clinical practice patterns. The detailed questions in part 3 investigated how the participants treated acute ST-segment elevation myocardial infarction (STEMI), acute non-ST-segment elevation myocardial infarction (NSTEMI), and unstable angina during the pandemic. The treatment of unstable angina with high-risk stratification is similar to that of NSTEMI. Therefore, in the questions related to unstable angina, we specified the condition of “unstable angina with low-moderate risk stratification”. The survey required approximately 25 minutes to complete.

### 2.3 Statistical Analysis

All statistical analyses were performed using SPSS 24.0 (IBM Corp, Armonk, NY, USA). Categorical variables are reported as the number and percentage. Continuous variables are expressed as the mean ± standard deviation (mean ± SD) for normally distributed variables, and the median (minimum–maximum) for non-normally distributed variables. Categorical variables were compared between groups by the Pearson chi-square test. A difference was accepted as significant at *p <* 0.05.

## 3. Results

### 3.1 Characteristics of the Survey Respondents 

Of the 300 clinicians invited, 211 (70.3%) completed the survey. The data were collected from physicians located in 22 provinces in China. Among the survey respondents, 82.5% were employed in tertiary hospitals, 52.1% reported more than 10 years of clinical cardiology practice, 61.1% had a subspecialty in coronary heart disease, and 46.4% were engaged in the interventional treatment of coronary heart disease (Table 1).

**Table 1.** **Characteristics of survey respondents (n = 211)**.

Characteristic		n (%)
Years in practice (years)		
	≤5	52 (24.6)
	6–10	49 (23.2)
	11–20	85 (40.3)
	>20	25 (11.8)
Classification of employed hospital		
	Primary general hospital	5 (2.4)
	Secondary general hospital	15 (7.1)
	Tertiary general hospital	174 (82.5)
	Cardiovascular hospital	17 (8.1)
Subspecialty (multiple choice)		
	Coronary heart disease	129 (61.1)
	Arrhythmias	107 (50.7)
	Congenital heart disease/structural heart disease	22 (10.4)
	Heart failure	62 (29.4)
	Hypertension	70 (33.2)
	Dyslipidemia	33 (15.6)
	Critical cardiovascular diseases	55 (26.1)
	Cardiovascular diseases without detailed subspecialty	53 (25.1)
	Other subspecialty	12 (5.7)
Subspecialty in interventional therapy (multiple choice)		
	Coronary artery intervention therapy	98 (46.4)
	Electrophysiology	85 (40.3)
	Cardiac device implantation	76 (36.0)
	Interventional therapy for congenital heart disease	18 (8.5)
	Interventional therapy for peripheral vascular diseases	4 (1.9)
	Other interventional therapy	3 (1.4)
	Not interventional physicians	56 (26.5)

### 3.2 Severity of the COVID-19 Pandemic in the Respondents’ Location

**Fig. 1.**
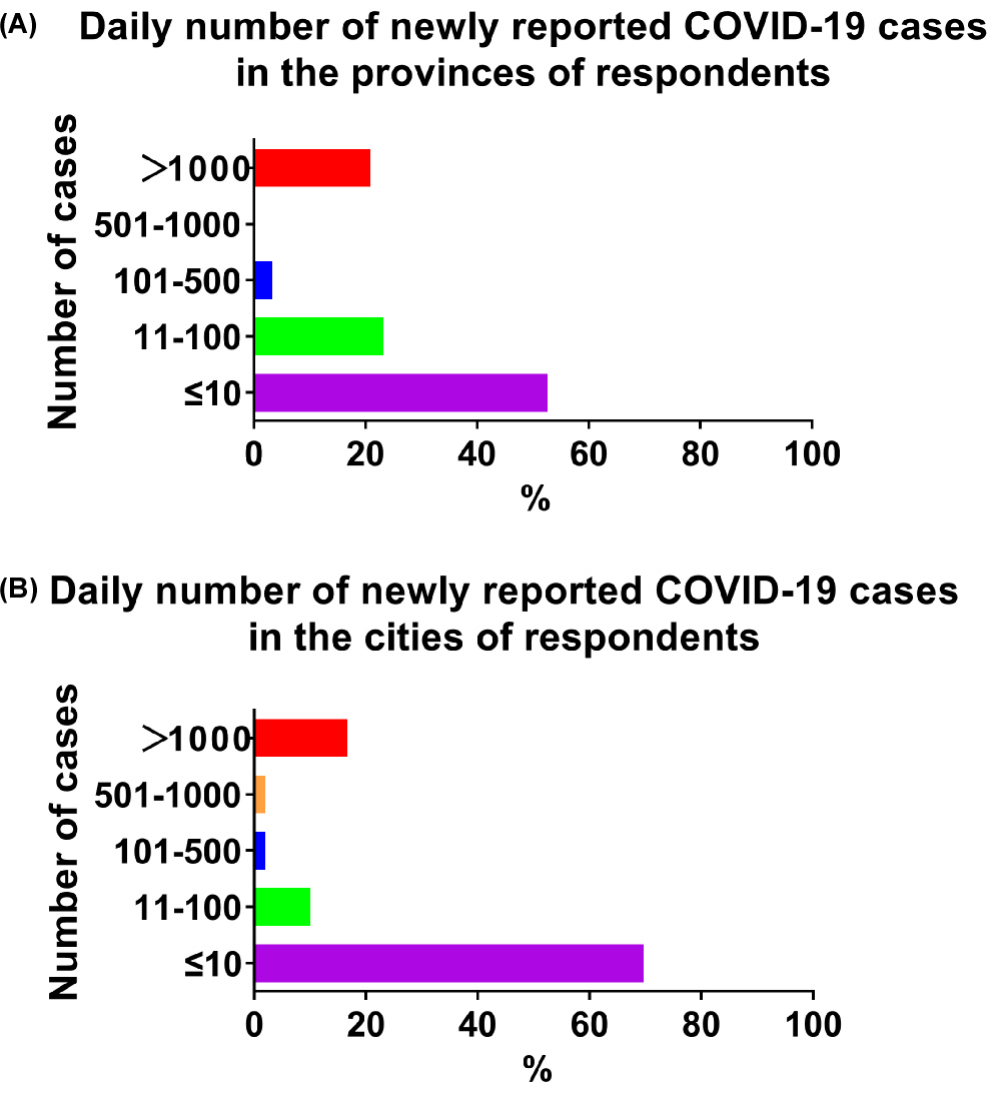
**Severity of the COVID-19 pandemic in the respondents’ location**. (A) Newly reported COVID-19 cases per day in the province of the respondents. (B) Newly reported COVID-19 cases per day in the city of the respondents.

As shown in Fig. 1, 20.9% of the respondents stated that the province in which they worked reported more than 1000 new COVID-19 cases per day in the week before responding to the survey, and 16.6% worked in a city where more than 1000 new COVID-19 cases were reported per day in the week before the survey.

### 3.3 Effect of the COVID-19 Pandemic on ACS Clinical Practice Patterns

#### 3.3.1 Effect of the COVID-19 Pandemic on the Number of ACS Inpatients and Outpatients 

**Fig. 2.**
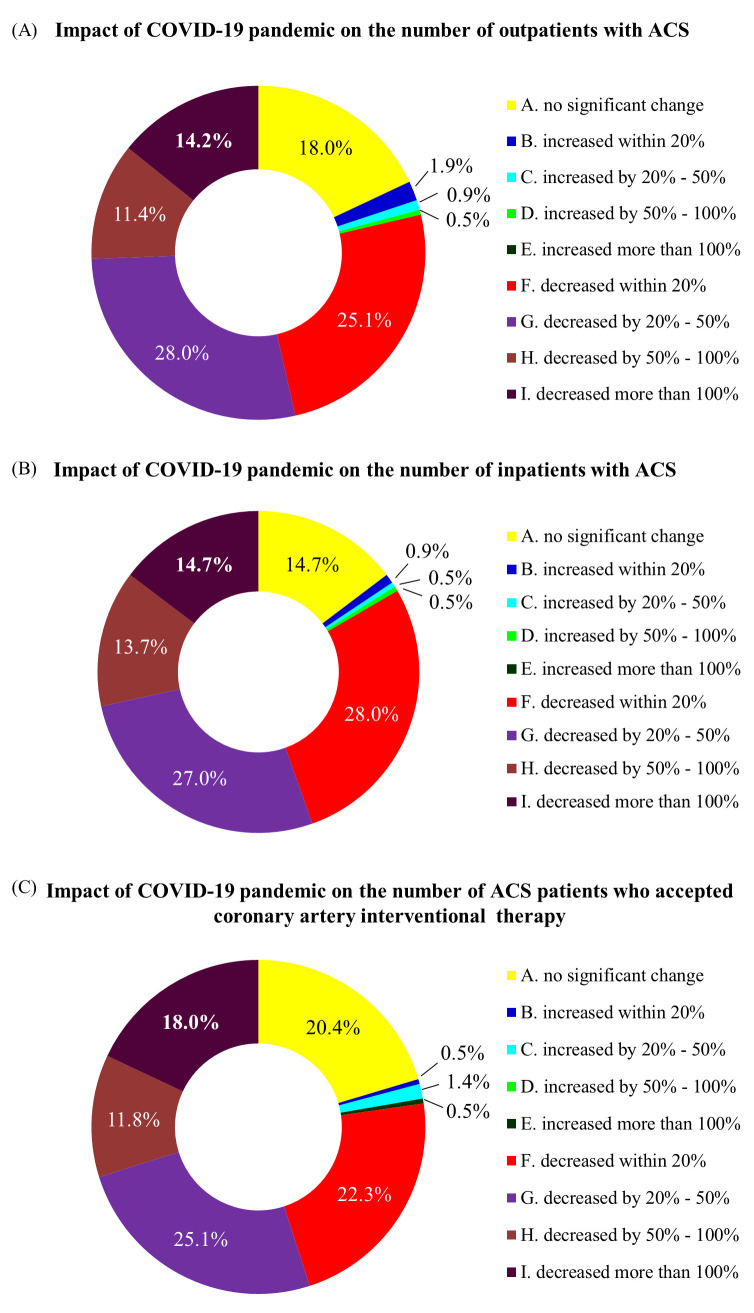
**Effect of the COVID-19 pandemic on the numbers of ACS inpatients and outpatients**. (A) Pandemic’s effect on the number of outpatients with ACS. (B) Pandemic’s effect on the number of inpatients with ACS. (C) Pandemic’s effect on the number of ACS patients who underwent coronary artery interventional therapy.

A small proportion of respondents stated that there was little change in the numbers of inpatients and outpatients with ACS, and an even smaller proportion stated that there was an increase in the numbers of these patients. The majority of respondents stated that there was a reduction in the numbers of ACS inpatients and outpatients during the pandemic (Fig. 2). There were similar results to the question about the number of ACS patients who underwent coronary artery interventional therapy.

Only 63 (29.9%) of the respondents worked in hospitals that had dedicated catheter rooms for the interventional treatment of ACS patients who also had COVID-19; 148 (70.1%) did not have access to dedicated catheter rooms in which to treat ACS patients with COVID-19.

#### 3.3.2 Effect of the COVID-19 Pandemic on STEMI Clinical Practice Patterns

Only 9.9% of the respondents stated that the COVID-19 pandemic did not affect their STEMI treatment practices, whereas the majority believed that the pandemic had varying degrees of effect on STEMI treatment (Table 2).

**Table 2.** **Cardiologists’ perceptions of how the COVID-19 pandemic affected ACS clinical practice patterns (n = 211)**.

		n (%)
Impact of COVID-19 pandemic on the treatment in STEMI		
	Almost no impact	21 (9.9)
	Mild impact	75 (35.5)
	Moderate impact	79 (37.4)
	Serious impact	36 (17.1)
Impact of COVID-19 pandemic on the treatment in NSTEMI		
	Almost no impact	24 (11.4)
	Mild impact	74 (35.1)
	Moderate impact	82 (38.9)
	Serious impact	31 (14.7)
Impact of COVID-19 pandemic on the treatment in unstable angina		
	Almost no impact	24 (11.4)
	Mild impact	75 (35.5)
	Moderate impact	90 (42.6)
	Serious impact	22 (10.4)

**Fig. 3.**
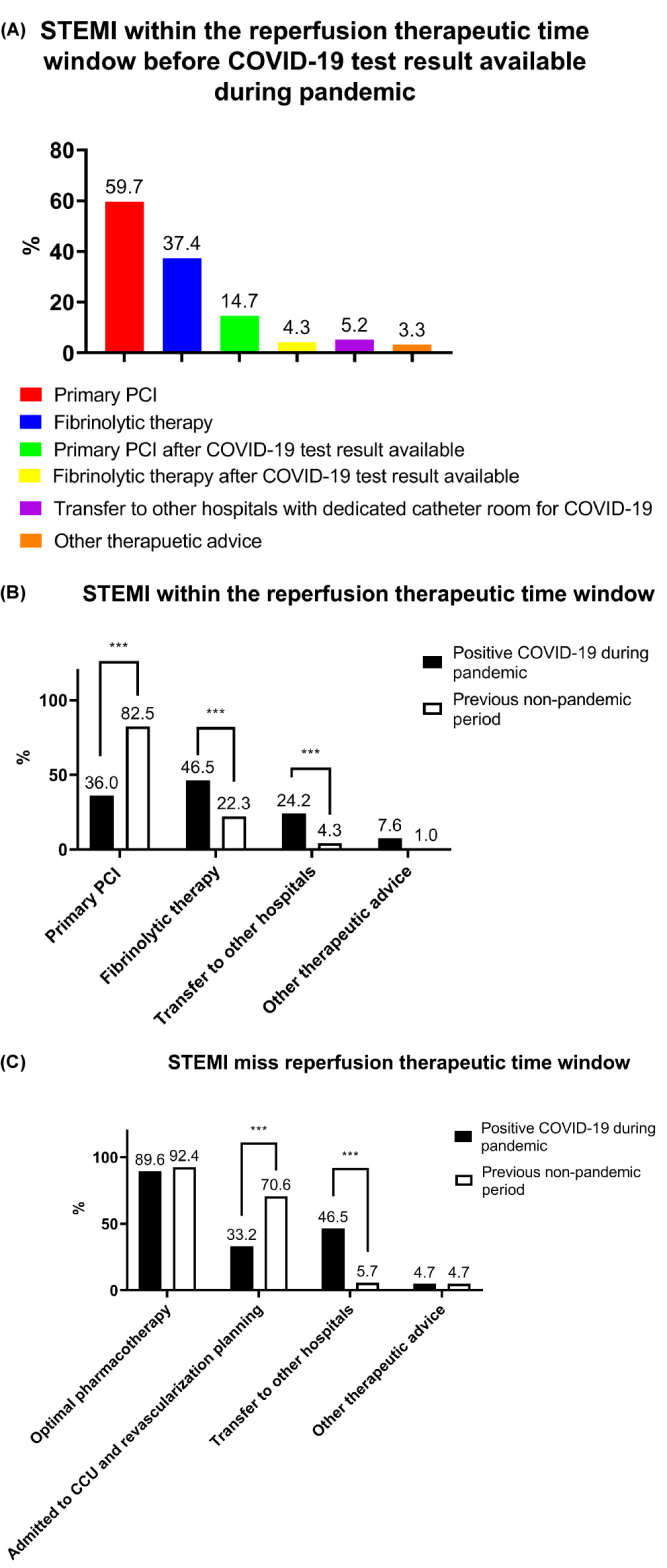
**Effect of the COVID-19 pandemic on STEMI clinical practice patterns**. (A) Respondents’ preferred reperfusion strategies for STEMI patients who did not have an available COVID-19 test result. (B) Respondents’ preferred reperfusion strategies for STEMI patients within the reperfusion therapeutic time window. (C) Respondents’ preferred treatment options for STEMI patients who had missed the reperfusion therapeutic time window. PCI, percutaneous coronary intervention; CCU, cardiac care unit; *** *p *< 0.001.

When asked “During the pandemic, what therapeutic strategies did you choose when a STEMI patient was within the reperfusion therapeutic time window but a COVID-19 test result was unavailable?”, nearly two-thirds (59.7%) of the respondents chose primary percutaneous coronary intervention (PCI), whereas 37.4% chose fibrinolytic therapy (Fig. 3A). A small proportion of respondents chose primary PCI or fibrinolytic therapy after the COVID-19 test result had become available, whereas 5.2% would transfer STEMI patients to other hospitals that had dedicated catheter rooms for COVID-19 patients.

For STEMI patients who were within the reperfusion therapeutic time window and were positive for COVID-19, 36.0%, 46.5%, and 24.2% of the respondents chose primary PCI, fibrinolytic therapy, and the transfer of patients to another hospital with a dedicated catheter room, respectively. These results were significantly different from the response to a similar question and option set, but under the assumption of the previous non-pandemic period (all *p <* 0.05) (Fig. 3B).

For STEMI patients who missed the reperfusion therapeutic time window and had a positive COVID-19 test result, 89.6%, 33.2%, and 46.5% of the respondents chose optimal pharmacotherapy, admit to the cardiac care unit and revascularization planning, and transfer to another hospital, respectively. Significantly fewer respondents chose revascularization planning in this scenario than in the period before the COVID-19 pandemic (*p *< 0.05). Compared with the previous non-pandemic period, a significantly greater number of respondents would choose to transfer the patient to another hospital with a dedicated catheter room (*p *< 0.05) (Fig. 3C).

#### 3.3.3 Effect of the COVID-19 Pandemic on NSTEMI Clinical Practice Patterns

Regarding the treatment of NSTEMI patients, 11.4% of the respondents stated that the COVID-19 pandemic had no effect on the treatment of NSTEMI, and the remainder thought the pandemic had varying degrees of effect (Table 2).

**Fig. 4.**
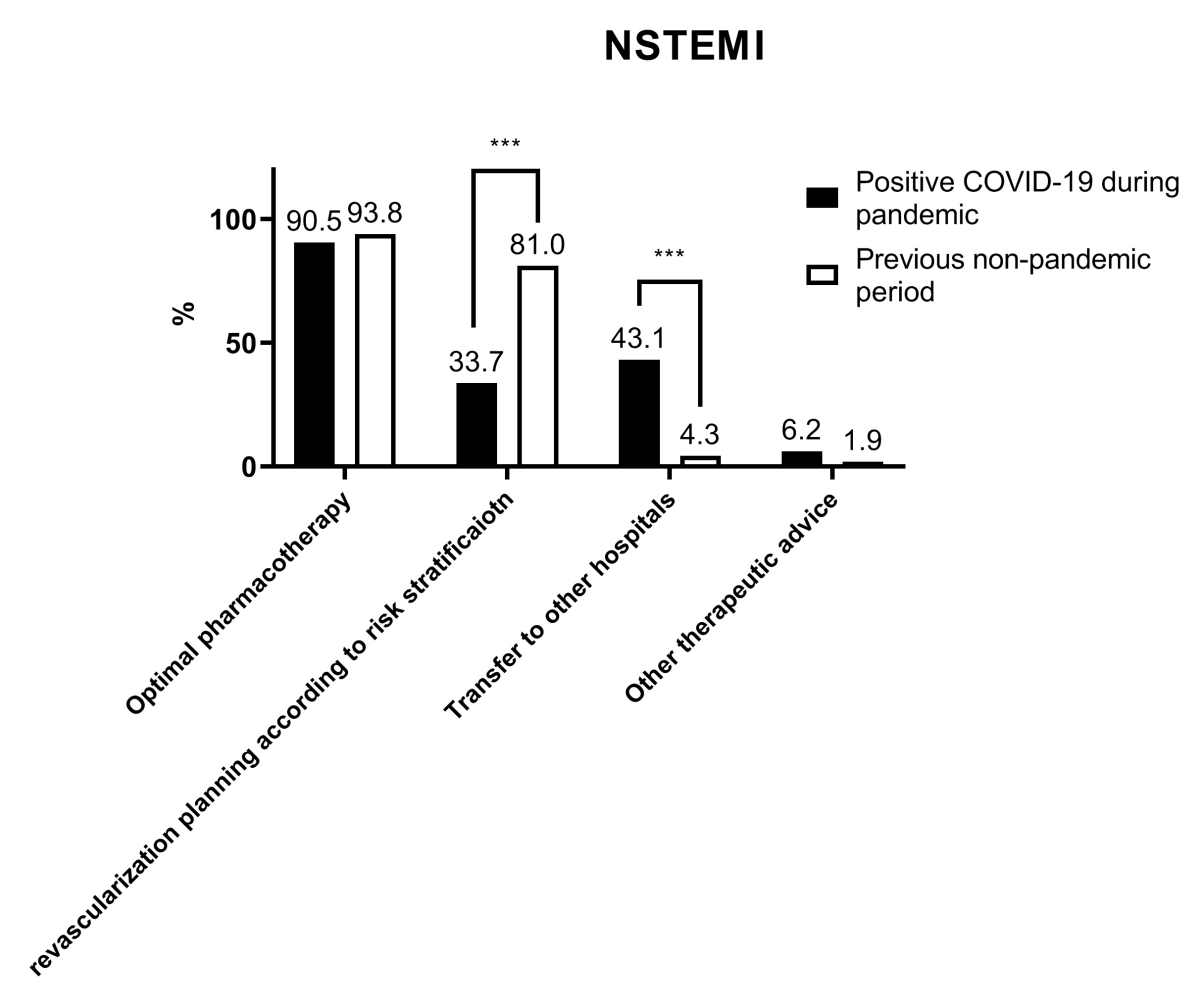
**Respondents’ preferred treatment for NSTEMI patients**. *** *p *< 0.001.

For NSTEMI patients with a positive COVID-19 test result, 90.5%, 33.7%, and 43.1% of the respondents chose optimal pharmacotherapy, revascularization planning according to risk stratification, and transfer to another hospital with a dedicated catheter room, respectively. The selection of revascularization planning and transfer to another hospital was significantly different from that in the non-pandemic period (all *p *< 0.05) (Fig. 4).

#### 3.3.4 Effect of the COVID-19 Pandemic on Unstable Angina Clinical Practice Patterns

A total of 88.6% of the respondents thought that the COVID-19 pandemic had varying degrees of effect on the treatment of unstable angina patients with low-moderate risk stratification (Table 2).

**Fig. 5.**
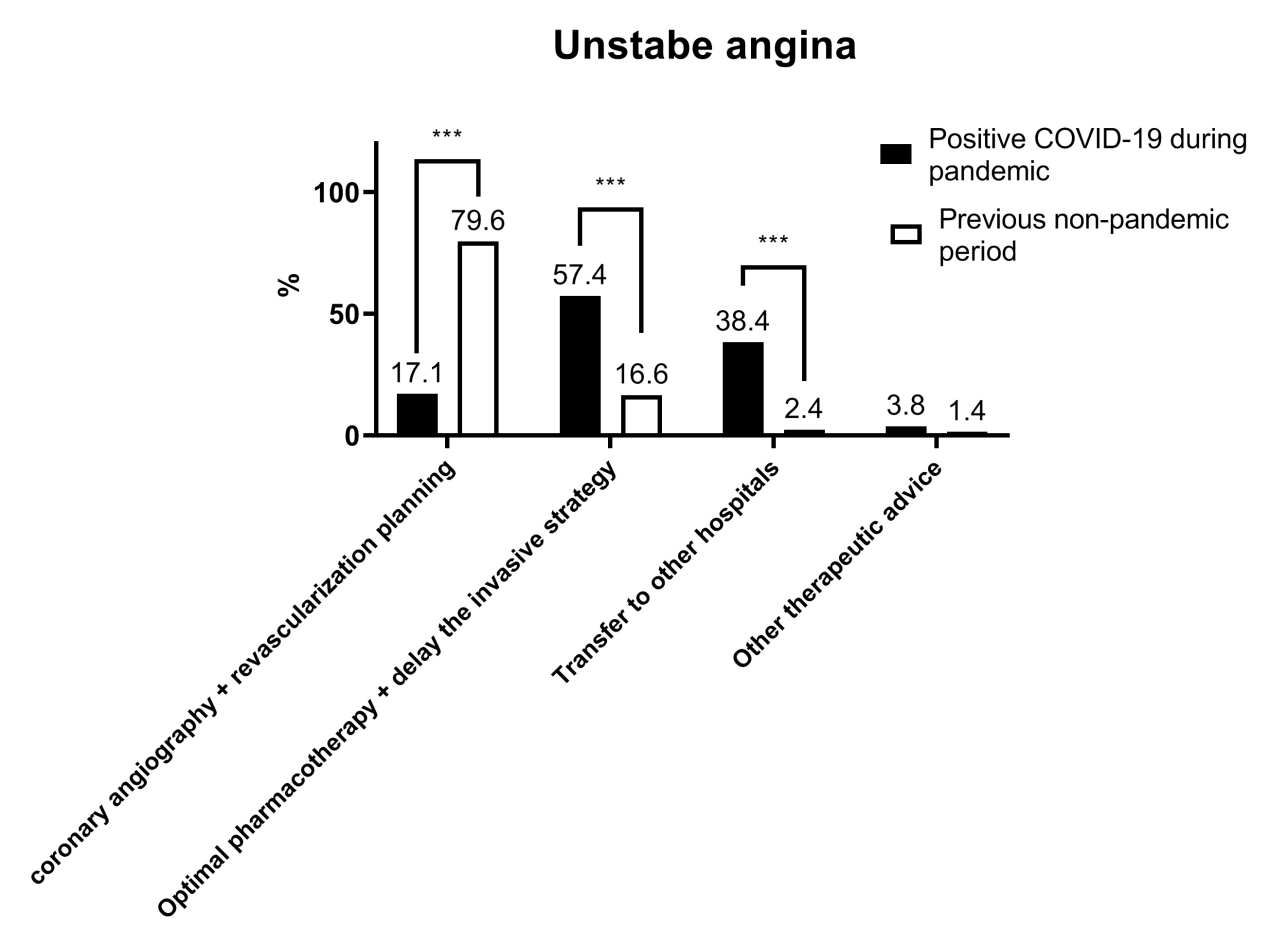
**Respondents’ preferred treatment for unstable angina patients with low-moderate risk stratification**. *** *p *< 0.001.

Regarding the treatment of unstable angina patients with low-moderate risk stratification who were positive for COVID-19, only 17.1% of the respondents chose the invasive strategy, whereas 57.4% preferred optimal pharmacotherapy and to delay invasive procedures; 38.4% chose transfer to another hospital with a dedicated catheter room. These results were significantly different from the response to a similar question and option set, but under the assumption of the previous non-pandemic period (all *p *< 0.05) (Fig. 5).

### 3.4 Relationship between Pandemic Severity and the Preferred Interventional and Fibrinolytic Therapies

As shown in Table 3, the selection of primary PCI and fibrinolytic therapy for STEMI patients within the reperfusion therapeutic time window but without a COVID-19 test result were significantly different among the participating cardiologists who had different daily numbers of newly reported COVID-19 cases in their cities (all *p *< 0.05). The selection of fibrinolytic therapy for STEMI patients within the reperfusion therapeutic time window who were positive for COVID-19 was also significantly different (*p* = 0.002).

**Table 3.** **Relationship between COVID-19 pandemic severity and the respondents’ preferred interventional and fibrinolytic therapies for ACS patients**.

	Daily number of newly reported COVID-19 cases in the cities of respondents					*p*-value
	≤10	11–100	101–500	501–1000	≥1000	
Option of primary PCI for STEMI patient within the reperfusion therapeutic time window but a COVID-19 test result was unavailable, n (%)	90 (61.2)	15 (71.4)	4 (100.0)	3 (75.0)	14 (40.0)	0.039
Option of primary PCI for STEMI patients within the reperfusion therapeutic time window and positive for COVID-19, n (%)	59 (40.1)	7 (33.3)	2 (50.0)	1 (25.0)	7 (20.0)	0.232
Option of revascularization plan for STEMI patients who missed the reperfusion therapeutic time window and positive for COVID-19, n (%)	55 (37.4)	4 (19.0)	2 (50.0)	1 (25.0)	8 (22.9)	0.249
Option of revascularization plan for NSTEMI patients and positive for COVID-19, n (%)	55 (37.4)	7 (33.3)	0 (0.0)	2 (50.0)	7 (20.0)	0.174
Option of revascularization plan for unstable angina patients and positive for COVID-19, n (%)	31 (21.1)	3 (14.3)	0 (0.0)	0 (0.0)	2 (5.7)	0.157
Option of fibrinolytic therapy for STEMI patient within the reperfusion therapeutic time window but a COVID-19 test result was unavailable, n (%)	46 (31.3)	6 (28.6)	1 (25.0)	2 (50.0)	24 (68.6)	0.001
Option of fibrinolytic therapy for STEMI patients within the reperfusion therapeutic time window and positive for COVID-19, n (%)	61 (41.5)	7 (33.3)	1 (25.0)	2 (50.0)	27 (77.1)	0.002

The characteristics of the participating cardiologists, including classification of employing hospital, years in cardiology practice, subspecialty, and subspecialty in interventional therapy, had no impact on their selection of interventional therapy and fibrinolytic therapy for ACS patients (all *p *> 0.05).

## 4. Discussion

The COVID-19 pandemic is having a marked effect on clinical practices around the world. In this multicenter survey of cardiologists in China, most stated that the COVID-19 pandemic had varying degrees of effect on their ACS diagnosis and treatment practices. During the pandemic, only 29.86% of the respondents had dedicated catheter rooms in their hospitals for the interventional treatment of ACS patients. Compared with the treatment choices in the period before the COVID-19 pandemic, the respondents’ choice of interventional treatments for ACS during the pandemic was reduced.

More than 80% of the respondents stated that during the pandemic there had been a substantial reduction in the numbers of ACS inpatients, outpatients, and patients undergoing catheter interventional therapy for ACS. This may have been because some ACS patients were worried about contracting COVID-19, and some patients with mild symptoms may have chosen to take medicine at home instead of going to the hospital. COVID-19 directly affects the development of thrombotic coronary obstruction and can lead to myocardial infarction through several mechanisms, such as inducing an inflammatory storm, a hypercoagulable state, or thrombosis, or through viral infiltration of myocardial cells or other immunologic mechanisms [13, 14, 15, 16, 17]. Previous clinical studies have demonstrated that ACS morbidity and mortality have significantly increased during the COVID-19 pandemic [5, 6, 18, 19]. The early treatment of ACS, especially for acute myocardial infarction, considerably benefits a patient’s prognosis. During the pandemic, the early transfer, early diagnosis, and early therapeutic interventions for ACS patients have been enormously challenging.

Although the COVID-19 pandemic currently affects the entire world, ACS presents with high morbidity and mortality and therefore should not be ignored. Moreover, a close relationship exists between ACS deterioration and COVID-19 virus infection at the pathophysiological level [20]. Respiratory failure caused by COVID-19 will aggravate the insufficiency of oxygen supply to the myocardium, thereby triggering and worsening myocardial infarction. Another critical mechanism implicated in the association between COVID-19 and ACS is the pro-inflammatory state. The inflammatory storm caused by COVID-19 plays a crucial role in eliciting an inflammatory pattern that may trigger ACS. Thus, the incidence of ACS in COVID-19 cases is correspondingly increased [21, 22]. A recently published meta-analysis found that ST-segment elevation can present in patients with COVID-19 regardless of pre-existing obstructive coronary artery diseases (CAD). COVID-19 patients without pre-existing obstructive CAD had increased diffuse ST-segment elevation and diffuse left ventricular wall-motion abnormality [23]. Saririan M *et al*. [24] reported an international case series of patients with confirmed COVID-19 infection and suspected STEMI, and none of the patients had obstructive coronary disease upon coronary angiography. Post-mortem histology demonstrated focal myocardial ischemia in one case, but there was no evidence of atherothrombosis or myocarditis [24]. Therefore, the COVID-19 pandemic has increased the incidence and deterioration of ACS in people with or without a confirmed history of CAD.

In patients with COVID-19 infection, the development of myocardial injury may lead to circulatory dysfunction and systemic imbalance of the oxygen supply, which can lead to the patient’s deterioration. Barman HA *et al*. [25] demonstrated that the incidence of myocardial injury could be as high as 24.7% among hospitalized COVID-19 patients. Compared with COVID-19 patients without myocardial injury, those with cardiac injury had a higher mortality rate and increased frequencies of requiring the intensive care unit (ICU), developing acute kidney injury, and developing acute respiratory distress syndrome [25]. Patients with pre-existing chronic coronary syndrome (CCS) have a significantly increased probability of myocardial injury and in-hospital death compared with patients without previous CCS, and patients with myocardial injury have a significantly increased mortality compared with patients without previous CCS. COVID-19 patients with coronary heart disease have higher rates of complications, in-hospital mortality, and frequency of renal replacement therapy than those without coronary heart disease [26, 27].

Coagulation derangement and thromboembolism are the major determinants of cardiovascular event involvement in COVID-19 patients. Severe COVID-19 is also associated with increased proinflammatory cytokine concentrations, which subsequently elicit coagulation activation and thrombin generation. Post-mortem findings in patients with COVID-19 have found microvascular thrombotic deposits in small vessels of the lungs and other organs [28]. These micro-macro thromboses can lead to hypoxemia and progress to a more severe status [29]. The hypercoagulable state associated with COVID-19 potentially increases the risk of thromboembolic complications [28, 30]. Tang N *et al*. [31] performed a retrospective study in China that included 449 patients with severe COVID-19 infection. It demonstrated that the prophylactic application of heparin significantly reduced mortality in patients with COVID-19-associated coagulopathy, especially in patients with increased concentrations of D-dimer. Schiavone M* et al*. [32] also found that in-hospital heparin administration was associated with a better chance of survival to hospital discharge. However, patients who received pre-hospitalization oral anticoagulation appeared to have more frequently developed acute hypoxemic respiratory failure and had a higher mortality rate. The exact mechanisms underlying the role of anticoagulant treatment and heparin in COVID-19 require further exploration.

We also found a significant decrease in the use of coronary interventions for STEMI, NSTEMI, and unstable angina during the COVID-19 pandemic. There may be multiple reasons for this phenomenon. In most cities around the world, medical care for cardiovascular events is provided by general hospitals. Treating ACS patients who have COVID-19 poses a risk of disease transmission to other patients and the medical staff. Owing to the distribution of healthcare resources, not all hospitals are equipped with dedicated cardiac catheter rooms for COVID-19 patients, which is likely why 36.0% of the respondents choose primary PCI for COVID-19-positive STEMI patients who were within the reperfusion therapeutic time window, whereas 46.5% choose fibrinolytic therapy for these patients. In the early stage of myocardial infarction, thrombolytic therapy is an important alternative to primary PCI. Fibrinolysis using urokinase, streptokinase, or alteplase remains the primary reperfusion strategy for STEMI in many developing countries [33, 34]. Therefore, while constrained by the COVID-19 pandemic, medical institutions without dedicated cardiac catheter rooms can prioritize fibrinolytic therapy for COVID-19-positive STEMI patients.

Early coronary interventional therapy, especially primary PCI for acute myocardial infarction, considerably improves ACS patients’ prognosis. Although the survey results showed that the proportion of cardiologists who choose fibrinolytic therapy for STEMI increased during the pandemic, patients will probably have a recurrent coronary event after thrombolytic therapy; thus, selective PCI is still needed for most STEMI patients who have previously undergone fibrinolytic therapy [35, 36]. The current expert consensus is that primary PCI remains the standard of care for STEMI patients during the COVID-19 pandemic at hospitals equipped with dedicated catheter rooms in which this treatment can be provided promptly [12]. From this perspective, the treatment of ACS during the pandemic has depended on not only the clinical competence of the physician but also the availability of medical resources. Countries and regions with sufficient resources can allocate dedicated catheter rooms for COVID-19-positive ACS patients.

## 5. Study Limitations

This study had several limitations. First, this was a multicenter clinician survey conducted in China, and differences in healthcare resource distributions and the COVID-19 pandemic severity among regions may have led to biased responses. Second, the clinical conditions of ACS patients often change. Thus, clinicians cannot always adhere to the diagnostic and therapeutic guidelines; rather, they must customize individualized treatment plans for different conditions. For this reason, the responses stated by the clinicians in the questionnaire may not completely reflect the actual management practices. Furthermore, the COVID-19 vaccines have played an important role in mitigating the pandemic and may profoundly impact the management of ACS patients. The main objective of this study was to investigate the perceptions of cardiologists regarding how the COVID-19 pandemic has affected the clinical practice patterns for ACS, and thus we did not collect information on vaccines in this survey. Additionally, the study findings need to be confirmed by further clinical studies.

## 6. Conclusions

The ongoing COVID-19 pandemic has had a profound effect on ACS clinical practice patterns. During the pandemic, the application of coronary interventional therapy decreased, whereas pharmacotherapy, especially fibrinolytic therapy for COVID-19-positive STEMI patients, was often stated as the best option by the participating cardiologists.

## Supplementary Material

Supplementary material associated with this article can be found, in the online version, at https://doi.org/10.31083/j.rcm2311362.
