# Supplementary material for: Effect of COVID-19 Pandemic on Acute Coronary Syndrome Clinical Practice Patterns: Findings from a Multicenter Clinician Survey in China
Source: Rev Cardiovasc Med. 2022 Oct 25;23(11):362. doi: 10.31083/j.rcm2311362 (PMC11269060; doi:10.31083/j.rcm2311362)
Supplement: Supplementary file 1 [file 2153-8174-23-11-362-s1.zip › Supplement Material.docx]

Dear colleague,

Welcome to the survey “Effects of the COVID-19 pandemic on clinical practice patterns for acute coronary syndrome”. The purpose of this survey is to help us better understand acute coronary syndrome treatment practices during the COVID-19 pandemic. The data collected in this survey will be kept anonymous. This survey includes demographic questions and hypothetical clinical questions. It should take you approximately 25 minutes to complete.

Part 1

1. In which province is your medical institution located?

2. What is the classification of the hospital you work in? [single choice]

A. Primary general hospital

B. Secondary general hospital

C. Tertiary general hospital

D. Cardiovascular hospital

3. How many years have you been practicing cardiology? [single choice]

A. ≤5

B. 6–10

C. 11–20

D. >20

4. What is your subspecialty in cardiology? [multiple choice]

A. Coronary heart disease

B. Arrhythmias

C. Congenital heart disease / structural heart disease

D. Heart failure

E. Hypertension

F. Dyslipidemia

G. Critical cardiovascular diseases

H. Cardiovascular diseases without detailed subspecialty

I. Other subspecialty

5. What is your subspecialty in interventional therapy? [multiple choice]

A. Coronary artery intervention therapy

B. Electrophysiology

C. Cardiac device implantation

D. Interventional therapy for congenital heart disease

E. Interventional therapy for peripheral vascular diseases

F. Other interventional therapy

G. Not an interventional physician

Part 2

6. What is the average daily number of newly reported COVID-19 cases in your **province** in the past week? [single choice]

A. ≤10

B. 11–100

C. 101–500

D. 501–1000

E. >1000

7. What is the average daily number of newly reported COVID-19 cases in your **city** in the past week? [single choice]

A. ≤10

B. 11–100

C. 101–500

D. 501–1000

E. >1000

Part 3

8. Compared with the previous non-pandemic period, how did the COVID-19 pandemic affect the number of **outpatients** with acute coronary syndrome in your hospital in the past month? [single choice]

A. No significant change

B. Increased by less than 20%

C. Increased by 20%–50%

D. Increased by 50%–100%

E. Increased more than100%

F. Decreased by less than 20%

G. Decreased by 20%–50%

H. Decreased by 50%–100%

I. Decreased more than100%

9. Compared with the previous non-pandemic period, how did the COVID-19 pandemic affect the number of **inpatients** with acute coronary syndrome in your hospital in the past month? [single choice]

A. No significant change

B. Increased by less than 20%

C. Increased by 20%–50%

D. Increased by 50%–100%

E. Increased more than100%

F. Decreased by less than 20%

G. Decreased by 20%–50%

H. Decreased by 50%–100%

I. Decreased more than100%

10. Compared with the previous non-pandemic period, how did the COVID-19 pandemic affect the number of **patients with acute coronary syndrome who underwent coronary artery interventional therapy** in your hospital in the past month? [single choice]

A. No significant change

B. Increased by less than 20%

C. Increased by 20%–50%

D. Increased by 50%–100%

E. Increased more than100%

F. Decreased by less than 20%

G. Decreased by 20%–50%

H. Decreased by 50%–100%

I. Decreased more than100%

11. During the COVID-19 pandemic, did your hospital set up dedicated catheter rooms for the interventional treatment of COVID-19-positive ACS patients? [single choice]

A. Yes

B. No

12. Regarding the treatment of acute ST-segment elevation myocardial infarction (**STEMI**), what level do you think the COVID-19 pandemic has affected you? [single choice]

A. Almost no effect

B. Mild effect

C. Moderate effect

D. Serious effect

13. During the pandemic, what therapeutic strategies did you choose when a STEMI patient was within the reperfusion therapeutic time window but a COVID-19 test result was unavailable? [multiple choice]

A. Primary PCI

B. Fibrinolytic therapy

C. Primary PCIafter the COVID-19 test result becomes available

D. Fibrinolytic therapy after the COVID-19 test result becomes available

E. Transfer to another hospital with dedicated catheter rooms for COVID-19 patients

F. Other therapeutic advice

14. During the COVID-19 pandemic, what therapeutic strategy did you choose when a STEMI patient was within the reperfusion therapeutic time window and had a positive COVID-19 test? [multiple choice]

A. Primary PCI

B. Fibrinolytic therapy

C. Transfer to another hospital with dedicated catheter rooms for COVID-19 patients

D. Other therapeutic advice

15. During the **previous non-pandemic period**, what therapeutic strategy would you choose if the STEMI patient was within the reperfusion therapeutic time window? [multiple choice]

A. Primary PCI

B. Fibrinolytic therapy

C. Transfer to other hospitals

D. Other therapeutic advice

16. During the COVID-19 pandemic, what therapeutic strategy did you choose when a STEMI patient had missed the reperfusion therapeutic time window and had a positive COVID-19 test? [multiple choice]

A. Optimal pharmacotherapy

B. Admit to cardiac care unit (CCU) / ward and revascularization planning

C. Transfer to another hospital with dedicated catheter rooms for COVID-19 patients

D. Other therapeutic advice

17. During the **previous non-pandemic period**, what therapeutic strategy would you choose if the STEMI patient had missed the reperfusion therapeutic time window? [multiple choice]

A. Optimal pharmacotherapy

B. Admit to CCU / ward and revascularization planning

C. Transfer to other hospitals

D. Other therapeutic advice

18. Regarding the treatment of acute non-ST-segment elevation myocardial infarction (**NSTEMI**), what level do you think the COVID-19 pandemic has affected you?  [single choice]

A. Almost no effect

B. Mild effect

C. Moderate effect

D. Serious effect

19. During the COVID-19 pandemic, what therapeutic strategy did you choose when a NSTEMI patient was positive for COVID-19? [multiple choice]

A. Optimal pharmacotherapy

B. Revascularization planning according to risk stratification

C. Transfer to another hospital with dedicated catheter rooms for COVID-19 patients

D. Other therapeutic advice

20. During the **previous non-pandemic period**, what therapeutic strategy would you choose for the NSTEMI patient? [multiple choice]

A. Optimal pharmacotherapy

B. Revascularization planning according to risk stratification

C. Transfer to other hospitals

D. Other therapeutic advice

21. Regarding the treatment of **unstable angina with low-moderate risk stratification**, what level do you think the COVID-19 pandemic has affected you?  [single choice]

A. Almost no effect

B. Mild effect

C. Moderate effect

D. Serious effect

22. During the COVID-19 pandemic, what therapeutic strategy did you choose when an unstable angina patient with low-moderate risk stratification was positive for COVID-19? [multiple choice]

A. Coronary angiography and revascularization planning

B. Optimal pharmacotherapy and delay invasive procedures

C. Transfer to another hospital with dedicated catheter rooms for COVID-19 patients

D. Other therapeutic advice

23. During the **previous non-pandemic period**, what therapeutic strategy would you choose for the unstable angina patient with low-moderate risk stratification? [multiple choice]

A. Coronary angiography and revascularization planning

B. Optimal pharmacotherapy and delay invasive procedures

C. Transfer to other hospitals

D. Other therapeutic advice
