# Supplementary figures and images for: Effect of COVID-19 Pandemic on Acute Coronary Syndrome Clinical Practice Patterns: Findings from a Multicenter Clinician Survey in China
Source: Rev Cardiovasc Med. 2022 Oct 25;23(11):362. doi: 10.31083/j.rcm2311362 (PMC11269060; doi:10.31083/j.rcm2311362)

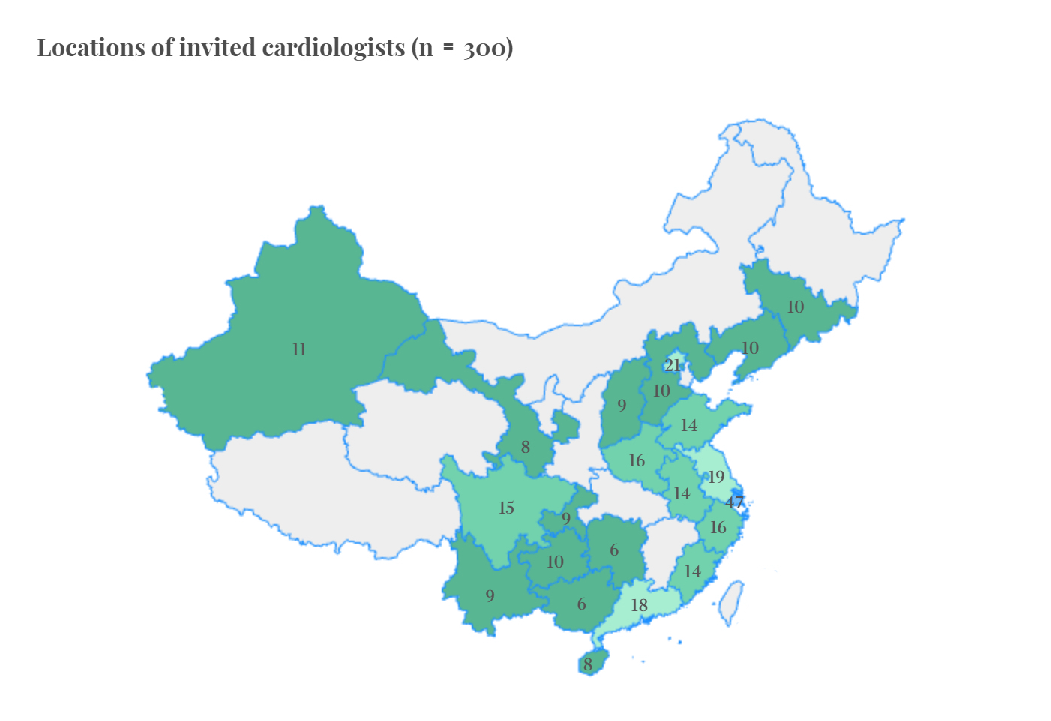

Supplement: Supplementary file 1 [file 2153-8174-23-11-362-s1.zip › Supplementary Fig. 1.tif]
